# Disruption of YY1-mediated super-enhancer–promoter looping drives transcriptomic changes during mammalian stem-cell aging

**DOI:** 10.21203/rs.3.rs-6531164/v1

**Published:** 2025-06-06

**Authors:** Luyang Sun, Brenna S. McCauley, Haiying Liu, Xueqian Chen, Weiwei Dang

**Affiliations:** 1 Huffington Center on Aging, Baylor College of Medicine, Houston, TX 77030, USA; 2 Single-Cell Center, CAS Key Laboratory of Biofuels, Shandong Key Laboratory of Energy Genetics and Shandong Institute of Energy Research, Qingdao Institute of Bioenergy and Bioprocess Technology, Chinese Academy of Sciences, Qingdao, Shandong, China; 3 University of Chinese Academy of Sciences, Beijing, China.

## Abstract

Stem-cell aging leads to a progressive decline in self-renewal and differentiation. How changes in chromatin architecture shape the gene-expression program underlying this loss of function remains incompletely understood. Here, we integrate transcriptomic, epigenomic, and Hi-C data from young and in-vitro-aged human mesenchymal stem cells (MSCs) to map super-enhancer–promoter (SE–promoter) loops and trace how they rewire during aging. SE target genes are enriched for Gene Ontology terms central to MSC identity and are disproportionately represented among age-regulated transcripts, suggesting that altered SE activity contributes to functional decline. YY1 is highly enriched at both promoters and SEs in young cells but is depleted from these loci in old cells. Loss of YY1 coincides with weakened Hi-C contacts, and YY1 knockdown in young MSCs recapitulates age-associated expression changes, especially among SE targets. Together, our results highlight YY1 as a key stabilizer of SE–promoter looping and gene-expression homeostasis during stem-cell aging.

## INTRODUCTION

Aging is a multifaceted process that culminates in organismal decline and death of all living organisms. While the phenotypic and physiological changes that occur during this process are well documented, the cellular and molecular processes that drive it remain poorly understood, though they include alterations to signaling pathways, chromatin, and mitochondrial function. Collectively, these alterations drive deleterious outcomes such as cellular senescence and stem-cell exhaustion ^1^. Stem cells are responsible for tissue regeneration and repair, and their diminished function contributes disproportionately to physiological decline ^2^. Ultimately, cell state is determined by its transcriptome, and gene expression changes with age in many tissues ^3–6^. Transcription is regulated at the level of chromatin ^7^. In particular, chromatin looping shapes genome organization and brings regulatory elements into direct contact with gene promoters ^8–10^. How chromatin organization changes during stem cell aging and how this impacts the transcription and functional decline of these cells has not been thoroughly investigated.

DNA looping underlies chromatin architecture. Distant loci are physically held together by cohesin rings, forming a loop ^11^. The endpoints, or anchor points, of these loops are determined by DNA binding proteins that self-associate and interact with cohesin, most notably CTCF, though other proteins also perform this function ^12–15^. The genome is folded into a series of nested loops, with short-range interactions occurring in the context of larger looped domains ^16^. In this paradigm, the topologically-associating domains (TADs) are often considered the basic unit of chromatin structure. TADs are self-interacting regions approximately 1 megabase long and loci within a TAD are insulated from interactions with external loci ^17^. Most chromatin loops are formed between sites within the same TAD, and disruption of TAD boundaries can promote contact between loci that are normally in distinct domains ^18^. At the other end of the spectrum, association and interaction between TADs is the basis for higher order chromatin structure and the 3D organization of the genome ^19^.

Previous studies on senescence-associated 3D genome reorganization have focused on high order structural changes at the level of compartment switching, TAD boundary shifts, and long- vs. short-range (or inter- vs. intra-TAD) interactions ^20–23^. While there is a correlation between compartment switching and gene expression changes in this context ^21^ in most cases, the relationship between chromatin architectural changes and gene expression remains unexplored. Interestingly, the depletion of CTCF disrupts TAD boundaries and alters chromatin interactions, similar to what is observed during senescence, albeit to a greater degree ^24,25^. Nevertheless, loss of CTCF has little effect on gene expression ^24,25^, suggesting that the disruption of TAD boundaries and insulation may not be major contributors to gene expression changes during aging.

Aside from compartmentalization into euchromatic and heterochromatic domains (compartments A and B, respectively), there is little evidence that higher order genome structure is a major determinant of gene expression ^26–28^. In contrast, enhancer-promoter looping is a strong determinant of gene expression ^29^. Numerous studies have implicated both enhancer “retargeting,” *i.e*., associating with a different promoter, and activation with gene expression changes during stem cell differentiation ^30–32^. In particular, super-enhancers, a class of enhancer characterized by long length and high levels of the activity-associated histone modification H3K27 acetylation (H3K27ac), are especially labile during differentiation ^31–33^. Super-enhancers tend to drive high levels of target gene expression and their target genes are essential for cell identity and key lineage-specific functions ^33^. Thus, dysregulation of super-enhancer target genes may contribute to the decline in stem cell function during aging. Changes in the enhancer-promoter regulatory landscape during aging and senescence have been the subject of several recent papers that have implicated this process with dysregulated gene expression and altered cell function ^34–41^. Critically, these works only considered chromatin accessibility and/or enhancer-associated histone modification enrichment changes and did not address how changing contacts between promoters and enhancers affect gene expression during aging.

How enhancer–promoter looping is physically achieved has been a long-standing question. Cohesin rings bridge these distant loci, as with TADs ^17,42^, but the identity of the structural protein that anchors most loops was only recently discovered. CTCF has long been known to localize to promoters and regulate gene expression from promoters to which it binds ^43^. However, global loss of CTCF does not affect the vast majority of enhancer–promoter looping, though it does mediate some long-range looping and guide promoter choice ^24,25,29,44^. Instead, YY1, a DNA binding protein with similarities to CTCF, mediates enhancer–promoter looping in embryonic stem cells and several human cell lines ^15^. Notably, YY1 is more highly enriched at promoters and enhancers than CTCF, and loss of YY1 both disrupts enhancer-promoter looping and gene expression ^15^. Thus, changes in the levels or genomic distribution of YY1 and/or CTCF during aging may have a profound impact on (super) enhancer-promoter looping and the gene regulatory landscape during stem cell aging.

To understand how genome organization changes during stem cell aging and what effect this has on gene expression and cell function, we focused on super-enhancer–promoter looping changes in *in vitro* aged human mesenchymal stem cells (MSCs). We identified changes in the super-enhancers that are active in young and old cells and in the strength of association between common super-enhancers and their target promoters during aging. As expected, the interactions between super-enhancer–promoter pairs are mediated by YY1 and not CTCF in these cells. During aging, there is an overall loss of YY1 binding to super-enhancer and promoter regions, and this loss of YY1 causes a dysregulation of gene expression. Thus, in mesenchymal stem cells, YY1 mediates promoter-super-enhancer looping, which stabilizes gene expression and loss of YY1 during aging dysregulates the expression of key lineage-specific genes, contributing to the functional decline of these cells as they age.

## RESULTS

### Large-scale chromatin 3-D structure is preserved during MSC replicative aging

**Figure 1:**
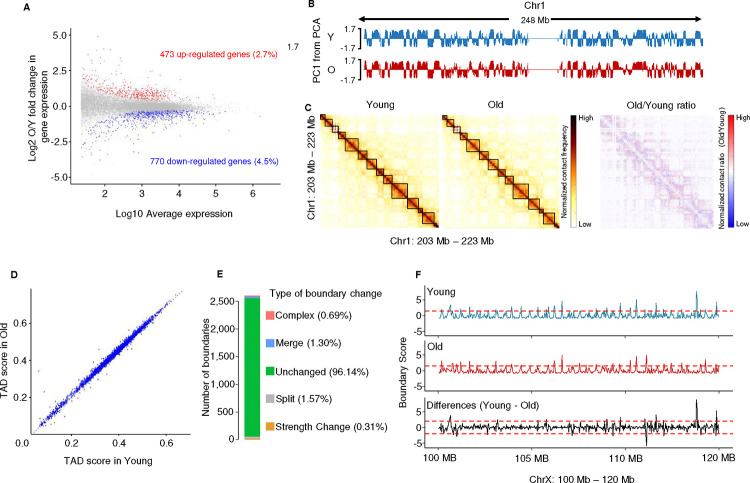
Global gene expression and long-range chromatin conformation changes during MSC aging A) Scatterplot of gene expression changes versus actual gene expression during MSC aging. Upregulated genes are indicated by red dots and downregulated genes by blue dots. B) Compartment analysis of Hi-C data showing the principal component 1 (PC1) value along chromosome 1 in young and old MSCs. C) Heatmaps showing Hi-C signal for young MSCs (Y, left), old MSCs (O, middle) and signal fold changes (right) at the genomic region chr1: 203,740,000–223,700,000. Topologically-associating domains (TADs) are indicated by black boxes; the black arrow shows a region with age-associated higher order chromatin structural changes. In the ratio plot, red indicates increased contacts in old cells. D) Scatterplot of global TAD scores calculated in old versus young MSCs. E) Distribution of the changes in TADs in old vs. young MSCs. F) TAD scores along a representative portion of chrX: 100 Mb-120 Mb, shown in young MSCs (top, blue), old MSCs (middle, red), and the difference (young-old, bottom, black). The dotted line represents the threshold (1.5 by default) to identify TAD boundary changes.

During aging, MSCs undergo a functional decline, characterized by decreased differentiation capacity, altered immunomodulatory signaling, slowed growth, and increased senescence ^45^. Perhaps unsurprisingly, this functional decline is accompanied by changes in gene expression. Examination of our previously published aging MSC RNA-seq data, which captured actively dividing cells with diminished differentiation potential ^46^, revealed that a total of 473 genes and 770 genes are significantly up- and downregulated, respectively, accounting for 7.2% of all expressed genes (Figure 1A). However, the chromatin changes that drive differential gene expression, and thus contribute to MSC aging phenotypes, have not been identified.

**Extended Data Fig. 1:**
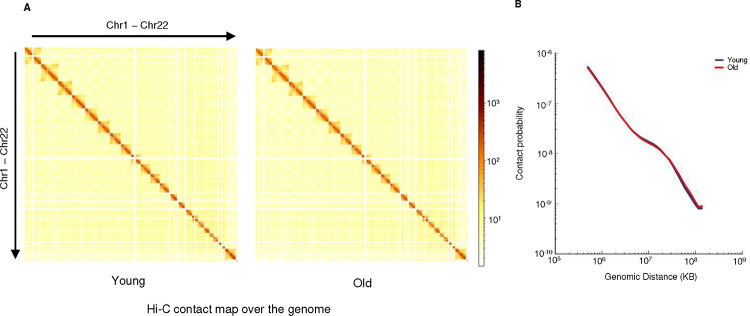
Global genome organization and identification of super-enhancer A) Heatmaps showing Hi-C signal over the genome (chr1-chr22) for young and old MSCs. B) Line plot showing the correlation between contact probability and genomic distance in young (blue) and old (red) MSCs.

To understand how genes are regulated during replicative aging, we first performed Hi-C ^47^ to characterize changes to the 3D genome structure that might impact gene regulation, specifically focusing on topologically associated domain (TAD) structure and compartment A/B distribution. However, the magnitude and the distribution of compartment A/B are highly similar between early and late passage MSCs (Figure 1B). Similarly, the vast majority of the TAD boundaries are conserved during outgrowth, with rare exceptions (Figure 1C, Extended Data Figure 1). Further analysis showed that the strength of TAD structure, indicated by TAD score ^48^ and mid-range contact frequency, also remains unchanged (Figure 1D, Extended Data Figure 1). Comparing TAD boundaries revealed that the majority (96%) are conserved in young and old MSCs, with the remaining merging, splitting, or undergoing strength changes (Figure 1E), in line with a previous report in senescent cells ^21^. As an example, TAD scores along a 20 Mb region of chromosome X are shown in Figure 1F, demonstrating generally stable TAD boundaries, with the splitting of one TAD into two in late passage MSCs. Thus, the relatively static higher order chromatin structure during MSC aging indicates that neither compartment switching nor altered TAD characteristics drive most age-associated gene expression changes.

Because global chromatin structure is largely conserved, we next examined fine-scale interactions that may be involved in regulating gene expression. For this, we turned to the previously published aging MSC histone modification ChIP-seq dataset ^46^. Here we focused on super-enhancers (SEs), a subset of enhancers that are longer and display higher levels of H3K27ac than other enhancer regions ^33^. These sites act as major cis-regulatory hubs that often drive the expression of genes associated with lineage-specific functions ^49^.

**Extended Data Fig. 2:**
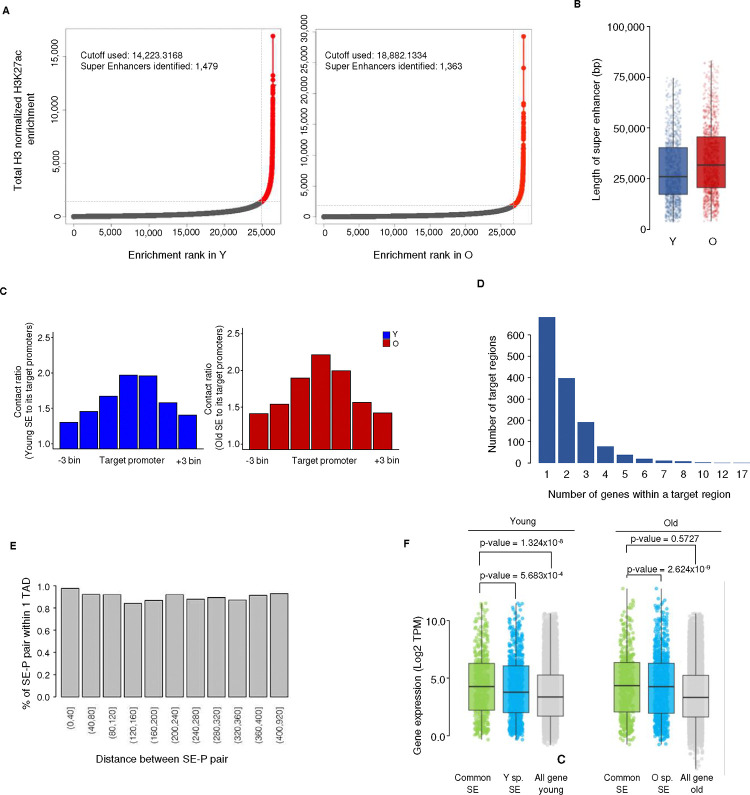
Expression and functional analysis of super-enhancer target genes A) Ranked plots showing the enrichment level of H3K27ac at enhancers in young (left) and old MSCs (right). Enhancer regions with extremely high levels of H3K27ac enrichment were identified as super-enhancers (red dots). B) Box plots showing the length of SEs in young and old MSC. C) Distribution of the average Hi-C contact ratio between SEs and their target promoters in young (blue) and old (red) samples. Hi-C contact ratio = contact frequency observed / contact frequency expected from a log-normal distribution. D) Distribution of the number of genes within a SE target region. E) Bar chart showing the percentages of SE–promoter pairs within 1 TAD grouped by the distance between the super-enhancer and its target promoter. F) Box plots showing the gene expression levels of common SE target genes (green), age-specific SE target genes (sky blue) and all genes (gray) in young (left) and old (right) MSCs. H) Bar chart showing the results of GO enrichment analysis of SE target genes in young and old MSC.

**Figure 2:**
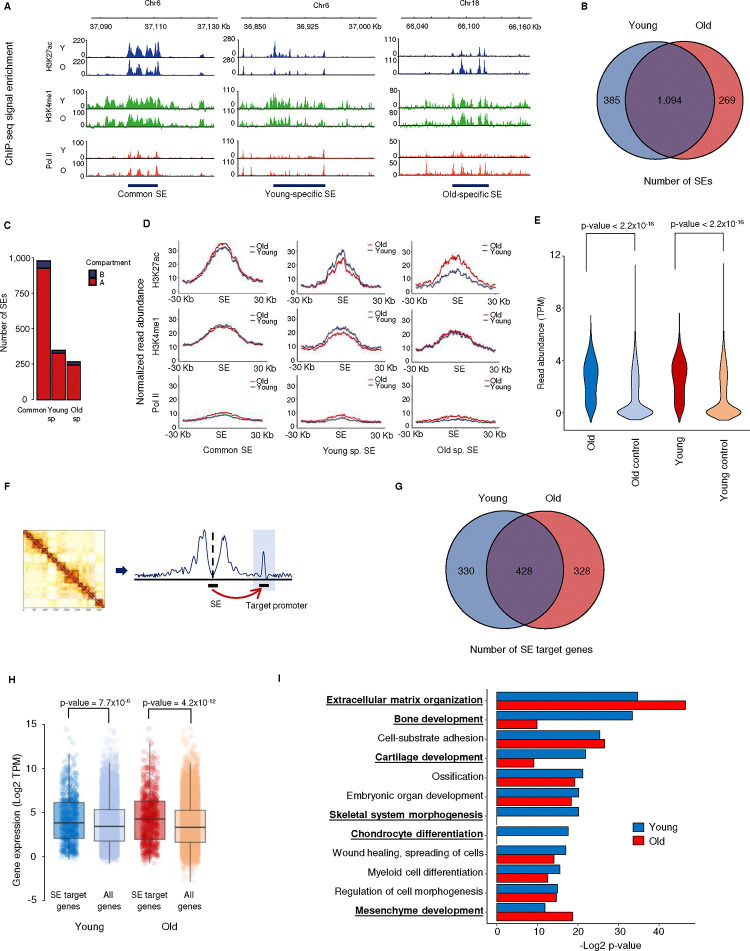
Super-enhancer target genes are associated with key facets of MSC function A) Signal tracks showing the distribution of H3K27ac (blue), H3K4me1 (green) and RNA Pol II (red) around representative super-enhancers (SEs). Common SEs are detected in both young and old MSCs, while young- and old-specific SEs are only identified in young and old samples, respectively. B) Venn diagram showing the overlap of SEs identified in young and old MSCs. C) Bar chart indicating the number of SEs located in Hi-C compartment A (red) and compartment B (blue). D) Metagene plots showing the occupancy of H3K27ac, H3K4me1 and RNA Pol II in common SEs, young-specific SEs, and old-specific SEs. The blue line represents young samples; the red line, old. E) Violin plots showing the production of eRNA by all SE regions (n =1478) in young and old samples, and their corresponding control regions (gray, n = 1,741). These represent the averages of 3 replicates of RNA-seq. F) Schematic of how the Hi-C results were used to predict SE target promoters. G) Venn diagram showing the overlap of SE target genes in young and old MSCs. H) Boxplots showing the gene expression level of SE target genes and all genes in young and old MSC. I) Bar chart showing GO categories enriched in SE target genes. Enrichment of SE targets in young MSCs is shown in blue, old in red. For p-values shown above boxplots, a two-sided Wilcoxon signed-rank test was used to determine significance.

Neither enhancers nor SEs had been defined from these datasets previously. We first defined active enhancers as non-promoter regions with significant enrichment of H3K27ac using the ROSE algorithm ^33^, and identified 26,422 in young MSCs (median length 1,670 bp) and 28,219 in old MSCs (median length 1,873 bp). A total of 23,153 (87.6%) were found in both young and old cells. SEs were defined as domains of enhancer clusters with extremely high levels of H3K27ac, identified using the ROSE method (Extended Data Figure 2). A total of 1,479 and 1,363 active SEs, median length 25–30 kb, were identified separately in early and late passage MSCs, respectively, with the majority of them common to both (Figure 2A, B, Extended Data Figure 2). As a part of the genome that is involved in active transcription, SEs are expected to be located in compartment A, as identified by Hi-C analysis. Indeed, most SEs (>90%) are found in this compartment (Figure 2C), indicating a positive correlation between SE identification and the Hi-C data.

Further characterization revealed that these active SEs are enriched with H3K4me1 and RNA polymerase II (Pol II; Figure 2A, D), in line with previous studies of active enhancers/super-enhancers ^50,51^. H3K4me1 is not just a marker of enhancers, but may also fine-turn their activity ^52^. In our data, the level of normalized H3K4me1 signal slightly correlates with presumed SE activity, *i.e*., young-specific SE regions contain higher H3K4me1 signal in young cells and old-specific SE regions have increased H3K4me1 levels in old cells (Figure 2D), further confirming the causative effect of H3K4me1 on enhancer activity. As expected, young- and old-specific enhancers are more enriched for H3K27ac in young and old MSCs, respectively (Figure 2D). To better characterize SE activity, we examined enhancer RNA (eRNA) expression, as it is well known that the degree of enhancer activity is reflected in the levels of eRNA ^53^. Indeed, the active SEs we identified on non-promoter regions have higher RNA-seq signal than the control regions (Figure 2E), indicating consistency between ChIP-seq based SE identification and their transcriptional activity.

In summary, our analyses indicate that the identified SEs are active and their overall activity changes with age, as indicated by H3K27ac, H3K4me1 and Pol II enrichment and eRNA expression, making them an ideal target for the investigation of loop-based enhancer–promoter regulation of gene expression during the aging process.

### Super-enhancers are associated with key functional genes in MSCs

The interactions between SEs and their target promoters are driven by chromatin looping and thus should be evident in Hi-C data. To identify putative target genes of the SEs, we converted the Hi-C matrix into virtual 4C maps using the H3K27ac-enriched SEs as anchor points (Figure 2F). Target regions were identified as 40-kb bins that have significantly higher contact frequencies with the SE than bins in a 2-Mb flanking region (Extended Data Figure 2). Genes that fall within the same 40-kb bin as the SE are by necessity overlooked in this analysis. All genes whose promoters fall within a target region were considered target genes of the SE in question. A total of 758 and 756 SE target genes were found in the young and old samples, respectively, with 428 of them in common (Figure 2G). The majority of SE target regions contain exactly 1 gene, irrespective of the distance between the two (Extended Data Figure 2).

SEs are widely accepted to regulate the expression of genes essential for maintaining cell identity and performing key cell-specific functions ^49^. In many cases, these diagnostic genes are relatively highly expressed ^54,55^. We asked whether either was true of the SE target genes identified in our analysis. Gene expression analysis showed that such genes have higher expression on average than all expressed genes in both young and old samples (Figure 2H), and that this is mainly driven by genes targeted by common SEs (Extended Data Figure 2), suggesting that SEs whose activity persists during aging drive higher expression than more labile, age-specific SEs. Furthermore, GO analysis of SE target genes in young cells identified enriched categories related to skeletal development, wound healing, and immune cell regulation, all of which are processes in which MSCs are key players ^45^ (Figure 2I). Interestingly, analysis of SE target genes in old cells revealed that many of these GO terms have decreased enrichment compared to young cells, particularly those related to bone/skeletal development; conversely GO terms related to extracellular matrix organization and mesenchyme development are more highly enriched in old cells (Figure 2I). This is consistent with the idea that the regions we identified are SEs that are crucial for maintaining MSC function.

**Figure 3:**
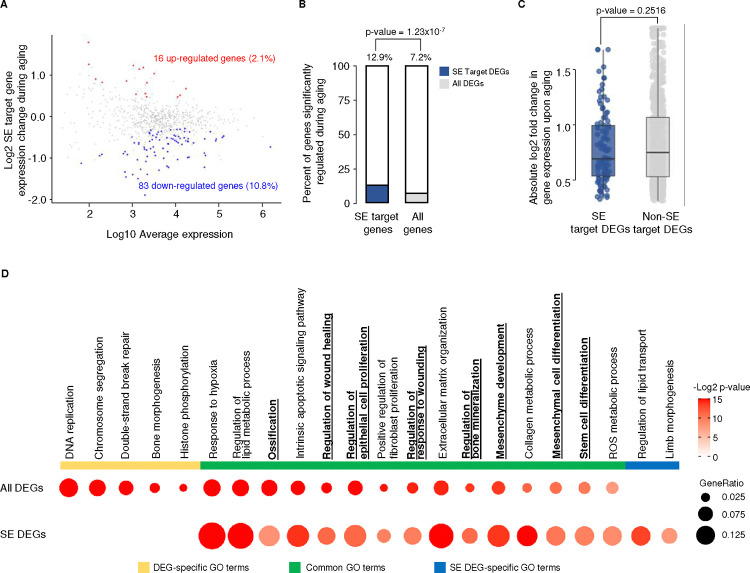
Super-enhancer target genes are more likely to be differently regulated during MSC aging A) Scatterplot showing gene expression fold change versus actual gene expression of SE target genes during MSC aging. Red dots, up-regulated genes; blue dots, down-regulated genes; gray dots, genes without significant gene expression changes. B) Bar charts showing the percentage of differentially expressed genes (DEGs) among SE target genes (left) and all expressed genes (right). The p-value was determined using Fisher's Exact Test. C) Boxplots of the absolute gene expression changes in SE target genes (blue, n = 99) and genes that are not targeted by an SE (gray, n = 1144). A two-sided Wilcoxon signed-rank test was used to determine significance. D) GO analysis of all DEGs and SE target DEGs. p-values were calculated by one-sided hypergeometric tests with the null hypothesis that the list contains more genes belonging to the GO cluster than expected. Gene ratio represents the proportion of genes in the GO cluster found in the list. The bold and underlined GO terms represent key MSC functions regulated by SEs with differential expression during aging.

As MSC function declines with age and SE target genes tend to be involved in key facets of MSC biology, we asked whether these genes are differentially regulated during aging (*i.e.*, are aging DEGs). Interestingly, although only a small fraction of SE target genes are aging DEGs, SE target genes have a significantly higher possibility of being regulated during aging, showing that genes regulated by SEs are involved in the MSC aging process (Figure 3A, B). It is noteworthy that among all aging DEGs, although not statistically significant, SE target DEGs have generally smaller expression changes (Figure 3C), which may be due to their higher basal expression levels. We next examined the functional categories of genes with aging-related changes in expression. GO term enrichment analysis showed that aging DEGs are enriched in many key pathways involved in stem cell differentiation, proliferation, and stress responses (Figure 3D). Interestingly, aging DEGs targeted by SEs are enriched in similar pathways, with even higher significance, and more focused on key lineage-specific functions of MSC, such as bone mineralization/ossification, wound healing, and mesenchymal cell differentiation (Figure 3D). As DEGs targeted by SEs are approximately 5 times as likely to be downregulated than they are to be upregulated during aging (Figure 3A), these GO categories are likely to represent processes that are less active in aged MSCs than in young. This is consistent with the idea that SEs regulate the expression of genes important for MSC function and suggests that disruption of this regulation contributes to the functional decline of MSCs during aging.

### YY1 anchors SEs to promoters and is redistributed during MSC aging

As SE target genes encode proteins important for MSC function and are overrepresented among aging DEGs, we wanted to further explore the interactions between SEs and their target promoters. CTCF and YY1 are both structural factors that mediate long range chromatin looping and orchestrate the regulation of cell lineage commitment, stem cell pluripotency, and single-cell level variation in gene expression ^56–58^, and both have been implicated in enhancer–promoter looping ^15,25,29,32,59^. As such, we sought to determine which of these factors plays the major role in SE-promoter looping in MSCs. We thus performed ChIP-seq for both factors in young and old MSCs and assessed their enrichment at regulatory elements and TAD boundaries.

**Extended Data Fig. 3:**
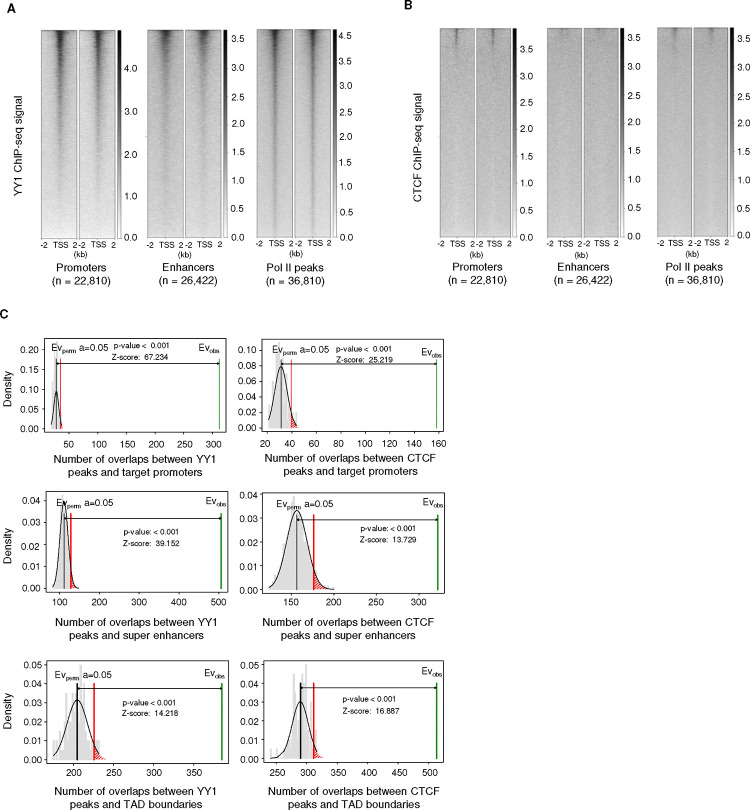
YY1 is more enriched than CTCF at promoter and enhancer regions. A) and B) Normalized ChIP-seq signal around promoter, enhancer and Pol II peaks for YY1 (A) and CTCF (B). C) Graphical representation of one-sided permutation tests for YY1 and CTCF peaks with the null hypothesis that the number of peaks that overlap with promoters, enhancers and TAD boundaries are equal to those found in random genomic regions.

**Figure 4:**
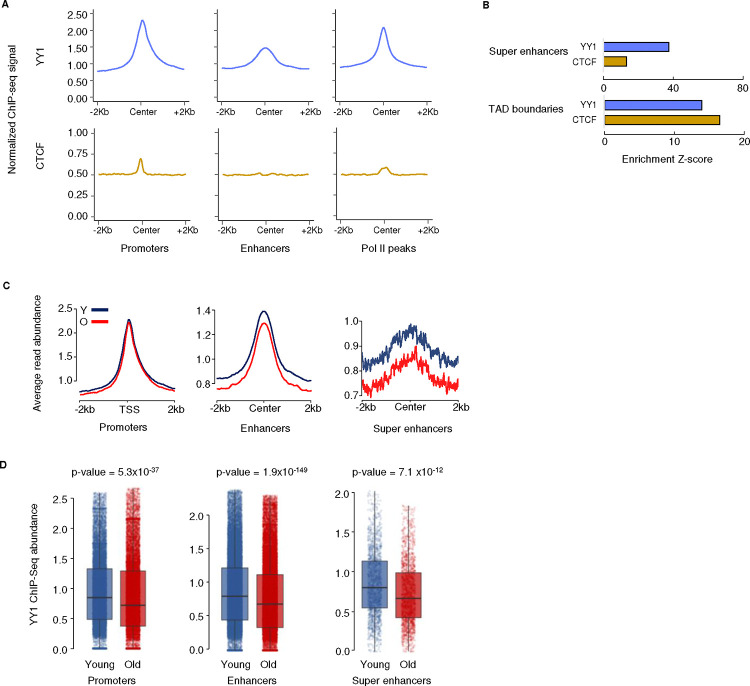
YY1 mediates enhancer–promoter looping and its level is decreased during MSC aging A) Metagene plots showing YY1 (blue) and CTCF (yellow) occupancy at promoters (n = 22,810), enhancers (n = 26,422) and RNA Pol II peak regions (n = 36,810) in young MSCs. B) Bar graphs showing the enrichment level of YY1 and CTCF signal at super-enhancers (SEs) and TAD boundaries in young MSC. Enrichment Z-score was calculated by a Permutation test, see Method for details. C) Metagene plots showing YY1 occupancy around promoters (n = 22,810), enhancers (n = 34,924) and SEs (n = 1,741) in young (blue) and old (red) samples. D) Boxplots of YY1 ChIP-seq abundance at promoters (n = 22,810), enhancers (n = 34,924) and SEs (n = 1,741) in young (blue) and old (red) samples. A two-sided Wilcoxon signed-rank test was used to determine significance.

To better understand the roles of YY1 and CTCF in gene regulation, we focused on their presence at enhancers, promoters, and sites where Pol II is bound. In young MSCs, YY1 is enriched in both active promoters and enhancers, and co-localizes with Pol II peaks, which represent hotspots of mRNA transcription (Figure 4A). However, while CTCF is enriched in promoter regions and colocalizes with Pol II, it is present at much lower levels than YY1, and is not enriched in enhancers (Figure 4A, Extended Data Figure 3), in agreement with the ‘enhancer release and retargeting’ theory ^29^. We next examined enrichment of YY1 and CTCF at the previously identified SEs. Perturbation tests showed that both proteins are significantly enriched at these loci, but YY1 is more highly enriched than CTCF (Figure 4B, Extended Data Figure 3). This trend is reversed at TAD boundaries, which show higher enrichment of CTCF (Figure 4B, Extended Data Figure 3), indicating that YY1 and CTCF have distinct roles at promoter-SE anchor regions and in making long-range constitutive structural contacts, respectively. Overall, these results demonstrate that YY1, not CTCF, is the prevalent binding protein at both promoters and enhancers in MSCs, supporting the idea that YY1 is more involved in mediating contact between promoters and enhancers in these cells.

As YY1 appears to be the major structural protein mediating SE-promoter interactions, we characterized the dynamics of the binding of this protein to these regions during MSC aging. There is a slight but significant decrease of YY1 binding to promoters in old MSCs (Figure 4C, D). Notably, enhancers, and particularly SEs, show a greater magnitude of YY1 loss with age than promoters do (Figure 4C, D). The loss of YY1 from promoters and (super) enhancers during MSC aging suggests a disruption in enhancer–promoter looping may occur, which could contribute to gene expression dysregulation in aged cells.

**Figure 5:**
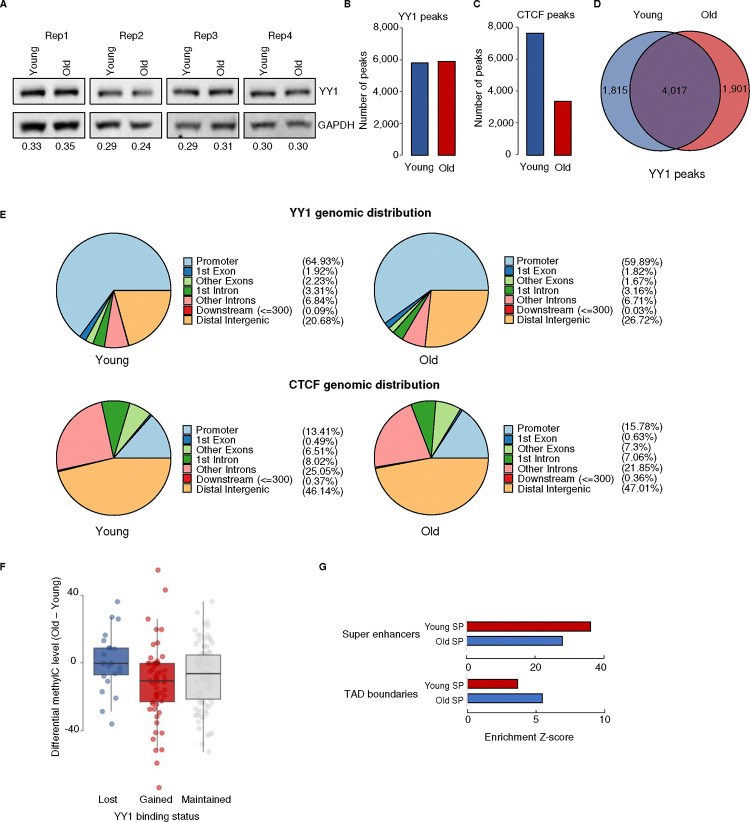
YY1 is redistributed to intergenic regions during MSC aging A) Western blot of YY1 and GAPDH in young and old MSCs. The numbers are the ratio of YY1/GAPDH in each condition. B) Bar chart showing the number of YY1 ChIP-seq peaks detected in young (blue) and old (red) MSCs. C), As (B), except for CTCF. D) Venn diagram showing the overlap of YY1 ChIP-seq peaks in young and old MSCs. E) Pie charts showing the genomic distribution of annotated YY1 and CTCF peaks in young and old MSCs. F) Boxplots showing the change in DNA methylation of the canonical YY1 binding site at YY1 peaks that are lost in old MSCs (lost YY1), gained in old MSCs (gained YY1), and maintained during MSC aging (maintained YY1). A two-sided Wilcoxon signed-rank test was used to determine significance. G) Bar graphs showing the enrichment level of young-specific (blue) and old-specific (red) YY1 ChIP-seq signal at super-enhancers (SEs, top) and TAD boundaries (bottom).

Loss of YY1 from promoters and SEs could reflect either reduced YY1 abundance or its redistribution across the genome. No changes in YY1 protein levels were detected in old vs. young MSCs by Western blotting (Figure 5A). In concordance with this, the number of YY1 peaks detected by ChIP-seq in young and old MSCs is quite similar (Figure 5B), which is in striking contrast to the sharp reduction in the number of CTCF peaks identified in aged vs. young MSCs (Figure 5C). However, comparing the overlap between the YY1 peaks revealed that only about two thirds of the peaks are present in both young and old cells, with almost one third unique to each age (1815 and 1901 peaks, respectively; Figure 5D), indicating that YY1 is redistributed along the genome during MSC aging. Indeed, analysis of the distribution of YY1 peaks in young and old MSCs reveals a shift away from promoters to distal intergenic regions of the genome (Figure 5E). This again differs from CTCF, the distribution of which is largely maintained in aged MSCs (Figure 5E). Thus, the loss of YY1 from promoters and SEs during MSC aging is due to the redistribution of this protein to new genomic loci.

We sought to understand what drives YY1 redistribution in old MSCs. HOMER analysis of transcription factor binding near young-specific, common, and old-specific YY1 peaks did not reveal clear differences in potential co-binding factors (not shown), suggesting that altered association with co-factors does not drive YY1 relocalization. YY1 itself binds DNA, canonically to the CGCCATnTT motif, and it is well established that CpG methylation within this site abrogates YY1 binding ^60^. We therefore compared the methylation status of YY1 binding sites at YY1 peaks that are lost, gained, or maintained during aging. While sites that lose YY1 during aging do not show a change in DNA methylation, there is a significant decrease in CpG methylation at YY1 binding sites at loci that gain YY1 peaks in old MSCs (Figure 5F). This suggests that the overall loss of DNA methylation characteristic of MSC aging ^46^ uncovers potential YY1 binding sites that were inaccessible in young cells, and these newly-available sites act as a sink, drawing YY1 away from promoters and SEs in old MSCs. Likewise, the global loss of CTCF binding during MSC aging (Figure 5C) may render YY1 sites at TAD boundaries accessible for YY1 binding, as suggested by the increased enrichment of YY1 at TAD boundaries in aged MSCs (Figure 5G). Thus, overall changes in the chromatin structure of old MSCs contribute to the redistribution of YY1 away from promoters and SEs during aging.

### Age-associated gene expression changes are substantially recapitulated by YY1 knockdown

**Extended Data Fig. 4:**
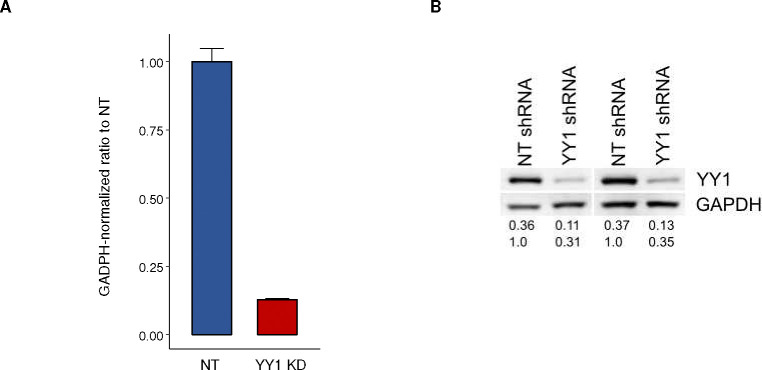
YY1 knockdown characterization A) qRT-PCR showing relative levels of *YY1* transcripts in control and knockdown cells. *GAPDH* was used for normalization. B) Western blot of YY1 protein levels in control and knockdown MSCs. The numbers are the ratio of YY1 to GAPDH in each condition.

**Figure 6:**
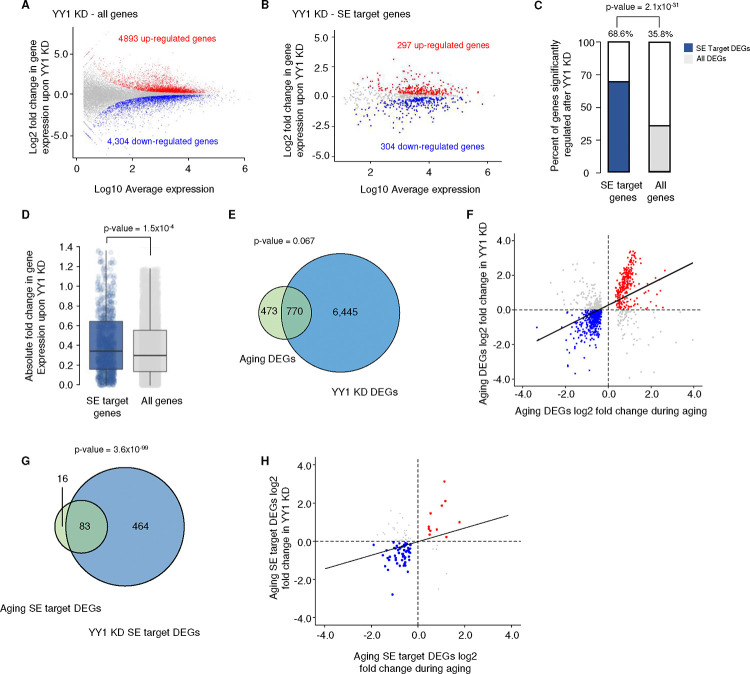
YY1 knockdown mimics MSC aging and has an outsized effect on SE target gene expression A) and B) Scatterplot showing RNA-seq profile of YY1 knockdown (KD) for all expressed genes (A) and the subset of SE target genes (B). Red dots, upregulated genes; blue dots, downregulated genes; gray dots, genes without a significant change in expression. C) Bar charts showing the percentage of differentially expressed genes after YY1 KD in SE target genes (left) and all expressed genes (right). The p-value was determined using Fisher's Exact Test. D) Boxplots of gene expression absolute fold change in SE target genes (blue, n = 1037) and all genes (gray, n = 12022) upon YY1 KD. E) Venn diagram showing the overlap of differentially expressed genes (DEGs) during aging and YY1 KD. F) Scatterplot showing the correlation of gene expression between YY1 KD and aging. Genes that were up-regulated (red) and down-regulated (blue) in both conditions are highlighted. G) Venn diagrams showing the overlap of SE target DEGs between YY1 KD and aging RNA-seq. H) Scatterplot showing the correlation of SE target gene expression between YY1 KD and aging RNA-seq. Genes that are up-regulated (red) and down-regulated (blue) in both conditions are highlighted. For the Venn diagrams, the p-values were determined using a hypergeometric test with the total number of genes equaling 12022.

The specific redistribution of YY1 away from *cis*-regulatory elements during MSC aging led us to further characterize the role of YY1 in regulating gene expression and its role in MSC aging. To this end, YY1 knockdown experiments were performed in early passage MSCs. This reduced *YY1* transcripts by approximately 85% and protein levels by ~65% (Extended Data Figure 4). Upon YY1 knockdown, dramatic gene expression changes were detected. A total of 4,893 significantly upregulated and 4,304 downregulated genes were found, constituting 35.8% of all expressed genes (Figure 6A, C). Analysis of SE target genes showed that 68.6% of them are differentially expressed after YY1 knockdown, including 297 upregulated genes and 304 downregulated genes (Figure 6B, C). Furthermore, SE target genes have a greater change in expression upon YY1 knockdown than genes that are not regulated by SEs (Figure 6D). This indicates that SE target genes are tightly regulated by YY1, and loss of YY1 results in an unstable gene expression profile.

A global comparison between the transcriptome profile of MSCs during replicative aging and YY1 knockdown revealed that 62% (770) of aging DEGs are also differentially expressed when YY1 is knocked down (Figure 6E). Furthermore, the expression changes of aging DEGs positively correlate with their gene expression changes upon YY1 knockdown (Figure 6F). Specifically, 80% of upregulated aging DEGs are also upregulated in YY1 knockdown samples and 68% of downregulated aging DEGs are also downregulated when YY1 levels are reduced, indicating that aging-related gene expression changes are at least partially reflected in the context of YY1 knockdown. The same trend is also found in SE target genes (Figure 6G, H), confirming that YY1 knockdown mimics the gene expression profile of MSC aging.

### YY1 stabilizes the expression of SE target genes via mediating super-enhancer–promoter interactions

So far, we have demonstrated that 1) YY1 is enriched in both SEs and their target gene promoters (Figure 4A, B); 2) the level of YY1 protein is reduced during MSC aging at enhancers and SEs (Figure 4C, D), and 3) YY1 is clearly involved in aging-related gene expression changes (Figure 6E, F). From this, and given its known role in enhancer–promoter looping ^15^, we hypothesized that YY1 plays a role in aging-related gene expression regulation by linking SEs and promoters through mid/long-range chromatin interactions.

**Extended Data Fig. 5:**
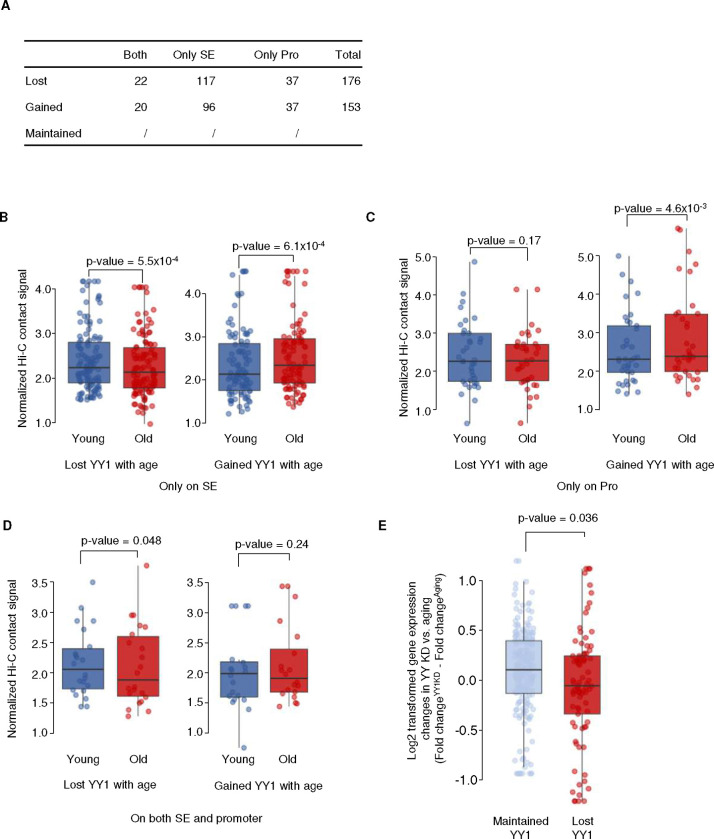
YY1 mediates SE-promoter loops and stabilizes gene expression of SE target genes A) Table indicating the number of super-enhancer (SE)-promoter pairs that fall into each category of gain/loss of YY1 during MSC aging for the analysis of YY1 function in SE-promoter looping. B-D) Boxplots showing Hi-C contact strength between SE-promoter pairs in young (blue) and red (old) MSCs under the following scenarios: SE-promoter pairs that only lost or gained YY1 on SEs (B); only on promoters (C); and on both SEs and promoters (D). E) Boxplots showing the log2-transformed gene expression differences between YY1 KD and aging in genes targeted by SEs that maintained YY1 (light blue) and genes targeted by SEs that lost YY1 upon aging (red). The sample size for the boxplots in B-D are described in A.

**Figure 7:**
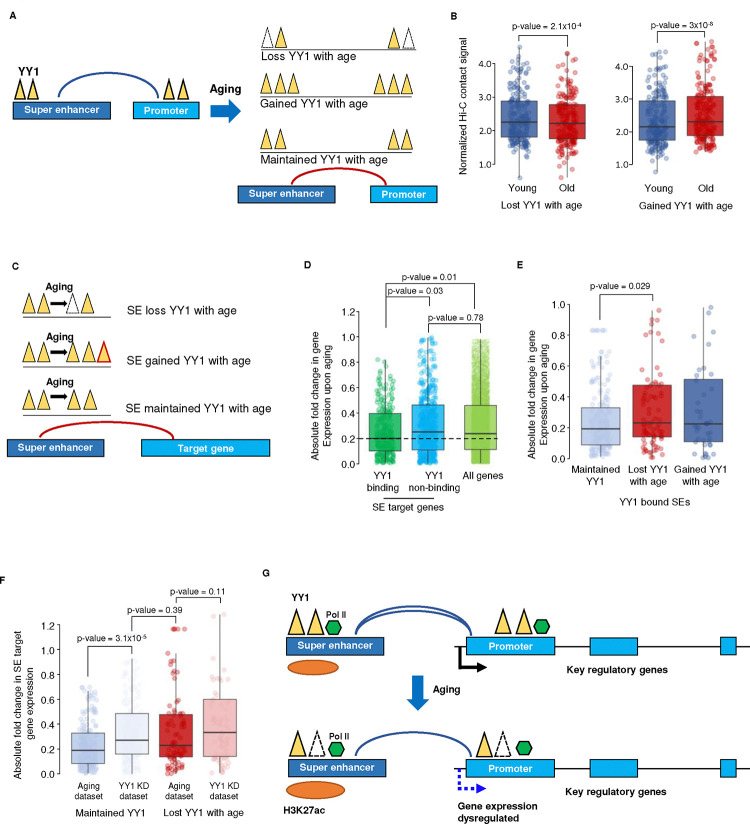
YY1 stabilizes the expression of SE target genes via mediating super-enhancer–promoter interactions A) Schematic showing the overall design of the analysis of YY1 function in mediating super-enhancer (SE)-promoter looping. SE-promoter pairs that lost or gained YY1 were identified; such pairs include those that lost or gained YY1 at only the promoter, at only the SE, or at both. B) Boxplots showing the level of Hi-C contact signal between SE-promoter pairs in young (blue) and old (red) MSC that lost (left, n = 223) or gained (right, n = 221) YY1 with age. C) Schematic showing the overall design of the analysis of YY1 localization to SEs affecting target gene expression. D) Boxplots showing the absolute log2-transformed fold change of gene expression during aging in YY1 binding SEs (dark green, n = 282), YY1 non-binding SEs (blue, n = 494), and all genes (light green, n = 17023). E) Boxplots showing the log2-transformed absolute fold change in expression during aging among genes regulated by SEs to which YY1 is bound, separated based on how YY1 changes with age at their cognate SEs, *i.e*., genes linked to SEs that maintained YY1 during aging (light blue, n = 196), SEs that lost YY1 with age (red, n = 85), and SEs that gained YY1 with age (dark blue, n = 44). F) Boxplots comparing the expression changes of genes linked to SEs that either maintained (blue, n = 196) or lost YY1 with age (red, n = 85) in aging vs. YY1 KD conditions. G) Schematic showing how YY1 functions to stabilize key regulatory gene expression through SE-promoter looping during MSC aging. For the p-values shown above the boxplots, a two-sided Wilcoxon signed-rank test was used to determine significance.

If YY1 functions to stabilize SE-promoter looping, we would predict that increased YY1 at either endpoint of the loop would promote contact between the SE and promoter, while decreased YY1 would have the opposite effect. To test this, we specifically identified two groups of SE-promoter loops, one with increased YY1 during aging and the other with decreased YY1 during aging at either the SE or promoter regions (Figure 7A, numbers for each category provided in Extended Data Figure 5) and examined their contact strength by Hi-C in young and old MSCs. Analysis of contact frequencies in the two groups revealed that SE-promoter loops with less YY1 during aging (“Lost YY1”) also have fewer Hi-C contacts between the SEs and their target promoters in old MSCs than in young. Similarly, SE-promoter loops with more YY1 protein in old cells (“Gained YY1”) also have more Hi-C contacts in the old sample (Figure 7B, Extended Data Figure 5). The concordance between the Hi-C contact frequencies and the levels of YY1 protein at SEs and their target promoter regions indicates that YY1 indeed mediates SE-promoter interactions.

Given the correlation between YY1 binding to SE-promoter loop anchor points and Hi-C contact strength, we next asked whether dynamic YY1 binding to SEs regulates expression of their target genes (Figure 7C). First, we compared expression changes between genes targeted by SEs to which YY1 is bound in either young or old cells (YY1 binding SEs) and genes targeted by SEs that are never enriched for YY1 (YY1 non-binding SEs). The results demonstrated that genes targeted by YY1 binding SEs have significantly lower gene expression changes during MSC aging (Figure 7D), indicating that the presence of YY1 at SE regions stabilizes the expression of SE target genes. Furthermore, genes targeted by YY1 non-binding SEs have similar gene expression changes as all expressed genes (Figure 7D), indicating that it is not the SE, *per se*, but the presence of YY1 at the SE that promotes gene expression stability.

As our previous analysis suggested a correlation between YY1 association with SEs and target gene expression stability, we next examined the consequences of YY1 loss at SE regions during MSC aging. SEs were divided into three groups based on the dynamics of YY1 during aging: YY1 was maintained, gained, or lost. SEs without YY1 enrichment in both young and old cells were excluded from analysis. Target genes of SEs that maintain YY1 with age have significantly lower gene expression changes than genes targeted by SEs that either lose or gain YY1 with age (Figure 7E). This suggests that the continuous presence of YY1 at SE-target promoter loops stabilizes gene expression during MSC aging.

Finally, if YY1 at SEs functions to stabilize expression of SE target genes, we predict that aging and YY1 knockdown will have similar effects on the expression of target genes of SEs that lose YY1 with age, while knockdown of YY1 will have a greater effect on expression of targets of those SEs that maintain YY1 levels during aging. Thus, we examined the expression of genes targeted by SEs with constant YY1 binding during aging and genes targeted by SEs where YY1 is lost during aging under these paradigms. The results show that genes targeted by SEs that maintain YY1 levels during aging have significantly higher gene expression changes when YY1 is knocked down than are seen during aging (Figure 7F, first two boxplots, Extended Data Figure 5). Conversely, there is no significant difference in gene expression changes during aging vs. upon YY1 knockdown among target genes of SEs that lose YY1 with age (Figure 7F, last two boxplots). Interestingly, the magnitude of expression changes of genes targeted by SEs that maintain YY1 with age during YY1 knockdown is similar to that of the expression changes of genes targeted by SEs that lose YY1 with age during aging (Figure 7F, second vs. third boxplot). Taken together, this confirms that loss of YY1 at SEs, whether during aging or upon YY1 knockdown, destabilizes expression of the genes targeted by these SEs, indicating that the presence of YY1 at SE regions stabilizes the expression of their target genes.

**Extended Data Fig. 6:**
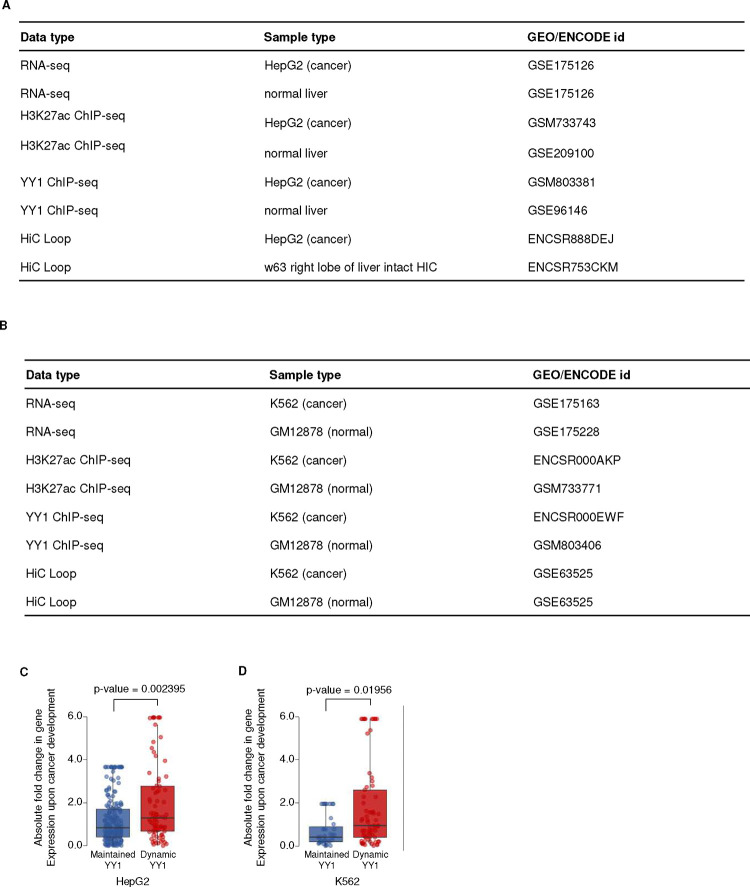
Analysis of public cancer datasets from ENCODE A and B) Dataset IDs used in the public cancer dataset analysis of hepatoblastoma (A) and myelogenous leukemia (B). C and D) Boxplots showing the absolute log2-transformed gene expression differences between SE target genes in normal tissue and transformed cells to which YY1 is (left, blue, maintained YY1 peak) or is not (right, red, dynamic YY1 peak) stably bound to the SE during oncogenic transformation. The transition to hepatoblastoma (normal liver vs. HepG2 cells; N=200 for maintained YY1 binding, N=80 for dynamic YY1 binding) is shown in C, and transformation to myelogenous leukemia (GM12878 vs. K562 cells; N=39 for maintained YY1 binding and N=69 for dynamic YY1 binding) in D.

To determine whether this is the case in other contexts, we assessed this process in two oncogenic transformations: liver tissue to hepatoblastoma (HepG2 cells) and B cells (GM12878) to myelogenous leukemia (K562 cells). From publicly available ENCODE datasets^61^, we analyzed gene expression and identified SEs and SE target genes in these cells as for MSCs (Figure S6A and B). During oncogenic transformation to both hepatoblastoma and myelogenous leukemia (Extended Data Figure 6), target genes of SEs that maintain YY1 binding during transformation have lesser changes in gene expression than SE target genes with which YY1 is not stably associated. This suggests that, as during MSC aging, YY1 functions to stabilize the interactions between SEs and their target genes and maintain consistent gene expression during oncogenic transformation.

Combined with our findings that active SEs in MSCs are linked to essential genes involved in core lineage-specific pathways such as mesenchymal cell differentiation, osteogenesis, stress responses, and self-renewal (Figure 2I and Figure 3C), our findings show that the presence of YY1 on active SEs promotes stable expression of key functional genes. During MSC aging, loss of YY1 on SEs is associated with weaker spatial contact between SEs and the target promoters, which contributes to the gene expression dysregulation that causes the phenotypic changes of MSCs undergo over the course of this process (Figure 7G).

## DISCUSSION

Our understanding of how altered chromatin structure impacts gene expression and cell function during aging is far from complete. Here, using a combination of Hi-C, ChIP-seq, and RNA-seq, we found that while TADs and higher order genome organization are largely preserved in aged MSCs, (Figure 1), there is substantial rewiring of SE-promoter loops that causes altered gene expression and likely contributes to the functional decline of these cells (Figures 2 and 3). In particular, the loss of the transcription factor YY1, which has been implicated in enhancer-promoter looping ^15^, from these sites correlates with both reduced SE-promoter contacts and dysregulated gene expression (Figures 4, 5, and 7), and loss of YY1 in young cells recapitulates age-associated transcriptomic changes (Figure 6). We propose that as YY1 is redistributed away from promoters and SEs during aging, these loops are disrupted, causing transcriptional dysregulation and contributing to the functional decline of MSCs.

Paradoxically, during aging and senescence, while TAD structure is largely preserved, both intra- and inter-TAD chromatin loops are perturbed (reviewed in Sun et al., 2018^62^). Indeed, in aged, non-senescent MSCs, higher-order chromatin structure is maintained, with TAD boundaries largely conserved between old and young cells and limited compartment switching (Figure 1, Extended Data Figure 1), similar to what as been reported in senescent fibroblasts ^20,63^. In fact, there appears to be a continuum of changes to higher-order chromatin structure from young to aged to early to “deep” senescent cells ^20 21 23^ (Figure 1), with regions of increased and decreased interactions becoming more discrete as senescence progresses. However, these supra-TAD-scale genome organization changes are not strongly correlated with gene expression changes during aging and senescence. Instead, during MSC aging, many of the altered chromatin interactions occur within TADs, suggesting that changes in short-range chromatin looping may regulate gene expression and cell function during aging.

A subset of intra-TAD chromatin interactions implicated in altered enhancer-promoter looping ^64^, which has clear implications for transcription. During MSC aging, there is substantial rewiring of SE-promoter loops, which is associated with altered expression of the SE target genes (Figures 2, 3, and 7). Previous work has shown global changes in enhancer activity during aging or senescence of various human and mouse cell types ^29,34–41^. These changes are associated with altered gene expression that contributes to aging or senescent phenotypes ^34,37,38,65^. Similarly, the altered SE regulatory landscape in aged MSCs is associated with dysregulated expression of genes involved in key MSC functions (Figure 3), linking altered (super) enhancer activity to transcriptional changes that impact aging stem cell function. Our use of Hi-C to identify the targets of enhancers and SEs is more robust than traditional, proximity-based methods ^32,66,67^, providing a direct link between changes in enhancers and differential gene expression in aging. While increased or decreased enhancer–promoter contact has been shown to modulate target gene expression in differentiating stem cells ^30,31^, this is the first study in which a similar phenomenon has been observed during aging.

In MSCs, as in several other cell types, significantly more promoters and (super) enhancers are bound by YY1 than by CTCF ^15^ (Figure 4, Extended Data Figure 3). Although YY1 mediates the majority of enhancer–promoter loops, CTCF drives a small portion of such loops, which are essential for critical cell functions, including differentiation and response to stimuli ^15,25 29^. Our results implicate YY1 in mediating SE-promoter loops in MSCs and suggest that its loss from these loops during aging disrupts SE-promoter association and dysregulates gene expression (Figures 4–7). Indeed, during oncogenic transformation, genes regulated by SEs that maintain YY1 have more stabile expression than those regulated by SEs with dynamic YY1 binding (Extended Data Figure 5), suggesting a widespread role for YY1 in stabilizing gene expression. These results are consistent with YY1 driving the majority of enhancer–promoter looping and implicating the loss of this protein in driving age- and disease-associated gene expression changes.

Significantly, the loss of YY1 from promoters and (super)-enhancers during MSC aging is driven by the relocalization of this protein to intergenic regions (Figure 5). Methylation of the canonical YY1 binding site inhibits YY1 binding ^60^. A genome-wide loss of DNA methylation is evident in aged MSCs, uncovering previously inaccessible YY1 binding sites, which can effectively titrate YY1 away from its normal binding sites (Figure 5) ^46^. More broadly, age-associated changes in DNA methylation may promote the mis-localization of YY1, generating novel, aberrant enhancer–promoter loops and dysregulating gene expression. Indeed, knock out of the DNA demethylases Tet2 and 3 in the developing mouse heart causes a global loss of YY1, disrupts higher order chromatin structure at these sites, and drives gene expression changes and abnormal cardiac development ^68^, demonstrating a clear link between YY1 mislocalization, genome organization, and dysregulated gene expression. Importantly, YY1 is required for quiescence of both hematopoietic and intestinal stem cells ^69,70^, and is necessary for satellite cell activation in response to injury ^71^, processes that are all perturbed during aging. Age-associated changes in DNA methylation and subsequent YY1 mislocalization may thus broadly contribute to the aging phenotypes of adult stem cells.

YY1 has additionally been implicated in neurodevelopmental disorders, neurodegeneration ^72^, cardiac development ^73^, and cancer ^74^. We posit that dysregulation of enhancer-promoter looping due to loss or gain of locus-specific YY1 binding, and subsequent gene expression changes, may contribute to the etiology of these diseases and heart defects. Indeed, YY1 is preferentially lost from enhancers in the neurodevelopmental disease Gabriele-de Vries syndrome, and target genes of such enhancers are dysregulated ^75^. The pleiotropic effects of YY1 in various cancers may also be due to a rewiring of the enhancer–promoter regulatory landscape, as novel enhancer-associated YY1 binding has been implicated in the progression and metastasis of hepatocellular carcinoma ^76,77^. YY1 is additionally critical for B cell development ^78^, and in pancreatic β cells, it is required for insulin expression, and loss of YY1 can drive the development of diabetes ^79^. The function of YY1 in mediating enhancer–promoter looping may provide a unifying mechanism for its pleiotropic roles.

This study is an important first step in understanding the role of altered chromatin loops in the aging process; it highlights that changes in relatively short-range chromatin interactions between SEs and promoters, rather than restructured higher-order genome organization, is associated with differential gene expression and functional changes to cells. It additionally suggests that the structural protein YY1 maintains promoter-SE looping and stabilizes gene expression during aging and directly links, for the first time, dysregulation of super-enhancer target genes and the functional decline of stem cells. We hypothesize that the rewiring of enhancer–promoter and other non-TAD forming chromatin loops during aging has a significant impact on transcriptomes, cell states and function, not just in MSCs, but more broadly across tissues. As assays like promoter capture Hi-C and Hi-ChIP evolve to require fewer cells, we anticipate more attention will be given to such questions.

## METHODS

### MSC cell culture

The source of MSCs and cell culture methods were described previously ^46^. Briefly, MSCs from human cord blood were isolated from a newborn white male. Cells were cultured in MSC growth medium (low glucose DMEM [Life Technologies 11885-084] with 10% FBS [Life Technologies 16000-044, lot #1314735] and 1× penicillin/streptomycin [Life Technologies 15140-122]) at 37°C with 5% CO_2_ and 3% O_2_. The growth medium was replaced every 4 days. Cells were split 1:4 when grown to ~70% confluence. Cells from PD 12 and PD 32 were used as young and old samples based on their differentiation potency ^46^.

### Western blotting

Total cellular protein was isolated from ~10^6^ MSCs in 100μL RIPA buffer with PFMS (Sigma 78830-5G) and 1× Halt Protease Inhibitor Cocktail (Fisher #PI78439). 10μg of protein was loaded per lane on a Bolt Bis-Tris Plus Mini Protein Gel 4–12% (Thermo Fisher, NW04122BOX) in MOPS running buffer. Proteins were transferred to an Immobilon-FL PVDF membrane (Fisher, IPFL00010) in Towbin buffer. Blots were blocked with LI-COR Intercept (TBS) Blocking Buffer (LI-COR, 92760001). Blots were incubated overnight at 4°C with primary antibodies diluted in LI-COR Intercept T20 (TBS) Antibody Diluent (LI-COR, 92765001) with 0.02% SDS. The YY1 antibody (abCam ab109237, lot GR3206274-4) was diluted 1:5000 and the GAPDH antibody (Invitrogen MA5-15738) was diluted 1:1000. The blots were then incubated with Goat anti-Mouse IgG (H+L) Highly Cross-Adsorbed Secondary Antibody, Alexa Fluor^™^ 680 (Thermo Fisher, A-21058) and IR Dye 800CW Goat anti-Rabbit IgG (LI-COR, 92632211) secondary antibodies, diluted 1:10,000 in LI-COR Intercept T20 (TBS) Antibody Diluent (LI-COR, 92765001) with 0.02% SDS for 1 hour at room temperature. Blots were imaged and analyzed using a LI-COR Odyssey system.

To assess YY1 levels during MSC aging, PD12 and PD32 cells were used. The YY1 knockdown experiment used control and YY1 knockdown cells at PD14.

### MSC YY1 knock down

shRNA-encoding lentiviral particles were used to knock down expression of *YY1* in MSCs as previously described ^46^. MISSION shRNA #TRCN0000019898 was used to knock down YY1 expression; the non-targeting shRNA #SHC002 (NT) was used as a control. Lentiviruses were produced in HEK 293T cells by co-transfecting the shRNA plasmids and plasmids encoding VSVG and ΔR8.9 (gifts from Zhixun Dou) using TransIT-VirusGen transfection reagent (Mirus Bio, MIR 6703) following standard protocols. Supernatants were collected 48 hours after transfection, centrifuged at 1,500×*g* for 10 min at 4 °C, and filtered through 0.45-μm filters. Lentiviruses were stored at −80 °C.

PD 10 MSCs were infected with *YY1* or NT shRNAs at 3 MOI in MSC growth medium with 6 μg/mL polybrene (Sigma #H9268-5G) for 6 hours and cultured in fresh MSC growth medium for 2 days under standard culture conditions. Successfully infected cells were selected for in MSC growth medium with 1μg/mL puromycin (Thermo Fisher #A1113802) for 6 days. RNA and protein were extracted from approximately 1×10^6^ PD 14 MSCs per preparation.

Successful knockdown of YY1 was confirmed by quantitative RT-PCR as described ^46^ and Western blotting. For qRT-PCR, following RNA extraction (Qiagen RNEasy miniprep kit, Qiagen 74104), 1 μg of RNA was reverse transcribed using the High-Capacity cDNA Reverse Transcription Kit (Thermo Fisher, #4368814). Reactions lacking the reverse transcriptase enzyme were included as controls. cDNA was diluted 1:30 for use in quantitative RT-PCR. 3.2 μL were used per 10 μL reaction, which contained 1× Fast SYBR green master mix (Thermo Fisher, #4385612) and 0.1 μM forward and reverse primers. Results were analyzed using the QuantStudio v1.3 package (Thermo Fisher). Primer sequences: *YY1*-F: AAGCCCTTTCAGTGCACGTT, *YY1-*R: CACATGTGTGCGCAAATTGA; *GAPDH*-F: CAGCCTCAAGATCATCAGCA, *GAPDH*-R: TGTGGTCATGAGTCCTTCCA. Western blots were performed as described above.

### ChIP-seq library preparation and data analysis

PD 12 and PD 32 MSCs were fixed with 1% formaldehyde ^80^. As described ^46^, fixed cells were lysed and chromatin was sonicated to approximately 400 bp. A total of 10 μL of cell lysate (equivalent to 100,000 cells) was used as initial input for both CTCF and YY1 ChIP. The True MicroChIP Kit (Diagenode, C01010130) and MicroPlex Library Preparation kit v.2 (Diagenode, C05010014) were used for ChIP-seq library construction, following manufacturer’s instructions. Antibodies used for CTCF and YY1 were AbCam #ab190237, lot GR3206274-4, and Active Motif #61311, lot 34614003, respectively. A total of 2 μL of cell lysate was used as input control. Libraries were sequenced at the Human Genome Sequencing Center at Baylor College of Medicine; CTFC ChIP-seq was run on an Illumina HiSeq 2500 and YY1 ChIP-seq on an Illumina NovaSeq.

In addition to YY1 and CTCF ChIP-seq, we analyzed H3K4me1, H3K27ac, and RNA polymerase II data from young and old MSCs ^46^. Raw reads were trimmed and mapped to the genome using fastp ^81^ and Bowtie2 version 2.4.2 ^82^. Peaks were called with MACS2 ^83^ using input (for YY1, CTCF, and RNA polymerase II) or H3 total (for histone modifications) as the control. Age-specific peaks were defined as peaks identified in young MSC but not in old MSC, or vice versa. Common peaks were defined as peaks at the same genomic locations that can be identified in both young and old MSC. Peak annotation was performed using the ChIPseeker R package ^84^ according to human reference genome hg19. Metaplots of YY1 and CTCF around promoters, enhancers, and RNA polymerase II peaks were made with deepTools2 ^85^. Enrichment analyses of YY1 and CTCF around enhancers, super-enhancers and promoters were performed using the regioneR R package ^86^ using regions around the YY1/CTCF peaks (± 2Mb) as background. Default parameters were used for all bioinformatic software/packages except noted. Enrichment Z-score was calculated by a Permutation test, representing the distance between the observed values and the mean of the expected values divided by the standard deviation of the expected values.

### *YY1* knockdown RNA-seq library preparation and RNA-seq data analysis

Total RNA extraction and RNA-seq library preparation were performed as described ^46^. Ribosomal RNAs were depleted from 100 ng of DNase-treated total RNA by NEBNext rRNA depletion kit (NEB, E6310). The NEBNext Ultra II Directional RNA Library Prep kit for Illumina (NEB, E7760) was used for RNA-seq library construction. The samples were sequenced on an Illumina NovaSeq at the Human Genome Sequencing Center at Baylor College of Medicine.

In addition to the YY1 knockdown RNA-seq performed here, we also analyzed RNA-seq data from young and old MSCs ^46^. All RNA-seq libraries were analyzed using the same pipeline. Low quality reads and sequencing adaptors were filtered using fastp version 0.12 ^86^. Clean reads were aligned to the reference genome (hg19) using HISAT2 version 2.2.1 ^87^. Gene-based read counting was performed using featureCounts version 2.0.3 ^88^. Differentially expressed genes were identified using DESeq2 version 1.34 ^89^ with the recommended workflow. FPKMs were calculated as described ^90^. GO analysis was performed using clusterProfiler version 4.0 ^91^. Default parameters were used for all bioinformatic software/packages except as noted. eRNA abundance from super-enhancers was analyzed using the method from ENCODE ^92^; for each SE, a corresponding control of region was generated by shifting 10 kb along the chromosome.

### Hi-C sequencing library preparation and data analysis

Hi-C sequencing was performed as described with modifications ^47^. About 1×10^6^ MSCs from both PD 12 and PD 32 were fixed with 1% formaldehyde and incubated at room temperature for 10 minutes, then quenched with 2.5 M glycine for 5 minutes. The cells were washed with PBS twice followed by brief centrifugation. MSCs were then lysed with Hi-C lysis buffer ^93^ and digested with MboI (NEB R0147S) overnight. Following DNA labeling, proximity ligation, crosslink reversal, and biotin pull-down, Illumina sequencing preparation steps were performed as previously described ^93^. The libraries were sequenced using the Illumina HiSeq 2500 sequencing platform at the Human Genome Sequencing Center at Baylor College of Medicine.

Hi-C data analysis was performed using ICE ^94^. An iterative multistep mapping strategy was used to map each end of the two paired-end reads independently. Each read was first truncated to 25 bp and mapped to the genome. Reads that did not map successfully were extended by 5 bp and remapped iteratively until the original read length was reached (100 bp). Aligned reads were then filtered based on the orientation, length, and concordance of the DNA restriction fragment. Filtered clean reads were binned along the genome at a 40 kb resolution and then normalized with the ICE method. The correlation between contact probability and genomic distance was done by first defining all possible genomic separations into logarithmical bins, then calculating the mean of Hi-C contact frequencies in each bin. Each chromosomal arm was analyzed separately, with interchromosomal reads excluded. PCA was performed using HOMER software at 100 kb resolution ^95^. TAD boundary identification was performed by InsulationScore ^48^. TAD scores were defined are the ratio of intrachromatin contacts versus the sum of intrachromatin contacts and interchromatin contacts ^96^. TAD boundary comparison between young and old MSC was conducted using the R package TADCompare v1.8.0 ^97^.

### Super-enhancer identification and target promoter prediction

Active super-enhancers (SEs) were identified from young and old MSC separately using the ROSE method ^33^ based on H3K27ac signal. Active enhancers were first identified by H3K27ac enrichment. Enhancers within 12.5 kb of each other were merged into a large domain to represent the whole span as one region. To identify super-enhancers, enhancers were ranked by the normalized abundance of H3K27ac. Based on the ranking plot, those with high levels of H3K27ac (above the inflection point with a tangent of 1) were defined as SEs. After initial identification, SEs were validated by checking the level of H3K4me1, RNA Polymerasee II, eRNA abundance, and age-specific SE activity changes in the defined regions.

The target promoters of SEs were predicted using Hi-C data. For each SE, a fitted local background model was built in its surrounding region (± 1Mb) using a log-normal distribution ^98^. By comparing the background model and actual Hi-C matrix, promoters within the 40 kb bins that had significant higher contact to the SEs (p-value < 0.001) were defined as the target of the SE. The p-value for each bin was determined by a two-sided Poisson test, with the null hypothesis that there are no Hi-C contact differences of the query bin - SE loop between actual Hi-C data and the background model. The contact frequency between the SE and its target bin were further compared to the surrounding regions of the target bin for validation.

### Analysis of YY1 function

YY1 function involved in SE and promoter looping during MSC aging were interpreted by the correlation of YY1 peak at the loop anchors and spatial contact frequency during aging. From all SE-promoter loops, we specifically identified those that lost or gained YY1 peaks during aging at either the SE or promoter anchor. Therefore, each group is a composed of 3 situations: YY1 only lost/gained at the SE region and no change at the promoter region, YY1 only lost/gained at the promoter region and no change at the SE region, and YY1 lost/gained at both promoter and SE region (Fig S5 A). For each SE-promoter loop in the defined group, its Hi-C contact frequency was extracted from the Hi-C matrix based on the genomic position. Then, the Hi-C contract frequency changes were compared between young and old MSC.

YY1 function in SE target gene expression regulation was interpreted by correlating the presence of YY1 at the loop anchor and gene expression changes during aging, then further validated by gene expression changes when YY1 was knocked down. Although YY1 is highly enriched in SEs, not all SEs contain YY1 peaks. SEs were separated into two groups: those to which YY1 is bound (YY1 binding SEs) and those to which it is not (YY1 non-binding SEs). The target gene expression changes of the two groups were compared, with all expressed genes in the transcriptome as reference. The YY1 binding SEs were further separated into three groups based on the changes of YY1 during aging: SEs that maintained YY1, SEs that lost YY1 with age, and SEs that gained YY1 with age. The expression of target genes of the three sub-groups were compared. Finally, gene expression changes during aging and gene expression under YY1 knock-down were compared between SEs that maintained YY1 and SEs that lost YY1 with age, to validate the function of YY1.

### YY1 motif scanning and binding site DNA methylation analysis.

Transcription factor motif enrichment in the 1kb regions flanking the YY1 peak summits was performed using the findMotifsGenome.pl script from the HOMER suite v4.11 ^99^ with default settings. To assess YY1 binding site methylation, publicly available whole-genome bisulfite sequencing datasets for young and aged MSCs were retrieved from the Gene Expression Omnibus (GEO accession number: GSE168063). Data preprocessing included the removal of redundant reads and the extraction of DNA methylation information, which was carried out using Bismark v.0.22.3 ^100^. CpG sites within the highest 0.1% of read coverage and those with fewer than 10 reads were excluded to minimize potential bias from overrepresentation and low coverage, respectively. Percentage difference of DNA methylation level between young and old MSC at each methylated CpG site was calculated, and then mapped to genomic regions characterized by differential YY1 binding, including regions with lost, gained, or unchanged YY1 association.

### Cancer datasets accessing and Hi-C informed SE analysis.

For myelogenous leukemia, RNA-seq (GSE175163, GSE175228), H3K27ac ChIP-seq (GSM733656, GSM733771), YY1 ChIP-seq (GSM935368, GSM803406) and in situ HiC (GSE63525) of K562 and GM12878 cells was obtained from the GEO and ENCODE databases. For hepatocellular carcinoma, RNA-seq (GSE175126, GSE175126), H3K27ac ChIP-seq (GSM733743, GSE209100), YY1 ChIP-seq (GSM803381, GSE96146), and intact HiC (ENCODE: ENCSR888DEJ, ENCSR753CKM) of HepG2 and normal liver cells were obtained. Differentially expressed genes, YY1 peaks, and super-enhancer regions were identified from the raw reads using the pipelines described above. For Hi-C data, contact loops were obtained from the processed files available from ENCODE/GEO.

Super-enhancers (SEs) were mapped in relation to their three-dimensional genomic context using Hi-C loop data. SEs overlapping with one anchor of a Hi-C loop were identified, and their corresponding spatial contact regions were predicted. Promoter regions within these SE-targeted areas were subsequently pinpointed using the EnsDb.Hsapiens.v86 database. Following the spatial analysis, SEs were categorized based on YY1 binding status: SEs that exhibited consistent YY1 binding from normal to cancerous cell types were grouped as the maintained YY1 binding category. In contrast, those with variable YY1 binding were placed in the dynamic YY1 binding category. Gene expression levels were compared between these 2 groups to elucidate the relationship between YY1 binding status at SEs and gene expression changes during the transition from normal to cancerous states.

## Extended Data

## Data Availability

The datasets generated during this study are available at the Gene Expression Omnibus (GEO) repository under GSE261011. RNA-seq, histone modification ChIP-seq, Pol II ChIP-seq and DNA methylation datasets of human mesenchymal stem cells were obtained from GEO (GSE156409). Additional cancer datasets used in this analysis are available from the ENCODE portal (encodeproject.org) and GEO portal (https://www.ncbi.nlm.nih.gov/gds), as described in Extended Figure S6 A and B.
